# Neural Dynamics of Target Detection via Wireless EEG in Embodied Cognition

**DOI:** 10.3390/s21155213

**Published:** 2021-07-31

**Authors:** Congying He, Rupesh Kumar Chikara, Chia-Lung Yeh, Li-Wei Ko

**Affiliations:** 1 Institute of Bioinformatics and Systems Biology, National Yang Ming Chiao Tung University, Hsinchu 30010, Taiwan; cyst0929.bt07@nycu.edu.tw; 2 Center for Intelligent Drug Systems and Smart Bio-Devices (IDS2B), National Yang Ming Chiao Tung University, Hsinchu 30010, Taiwan; 3 Jane and John Justin Neurosciences Center, Cook Children’s Health Care System, Fort Worth, TX 76104, USA; rupesh.kumarchikara@uta.edu; 4 Department of Bioengineering, University of Texas at Arlington, Arlington, TX 76109, USA; 5 Information and Communications Research Division, National Chung-Shan Institute of Science and Technology, Taoyuan 325, Taiwan; austinyeh@msn.com; 6 Department of Electrical and Computer Engineering, and Brain Research Center, National Yang Ming Chiao Tung University, Hsinchu 30010, Taiwan; 7 Drug Development and Value Creation Research Center, Kaohsiung Medical University, Kaohsiung 807, Taiwan

**Keywords:** wireless EEG, brain computer interface, embodied cognition, target detection, attention

## Abstract

Embodied cognitive attention detection is important for many real-world applications, such as monitoring attention in daily driving and studying. Exploring how the brain and behavior are influenced by visual sensory inputs becomes a major challenge in the real world. The neural activity of embodied mind cognitive states can be understood through simple symbol experimental design. However, searching for a particular target in the real world is more complicated than during a simple symbol experiment in the laboratory setting. Hence, the development of realistic situations for investigating the neural dynamics of subjects during real-world environments is critical. This study designed a novel military-inspired target detection task for investigating the neural activities of performing embodied cognition tasks in the real-world setting. We adopted independent component analysis (ICA) and electroencephalogram (EEG) dipole source localization methods to study the participant’s event-related potentials (ERPs), event-related spectral perturbation (ERSP), and power spectral density (PSD) during the target detection task using a wireless EEG system, which is more convenient for real-life use. Behavioral results showed that the response time in the congruent condition (582 ms) was shorter than those in the incongruent (666 ms) and nontarget (863 ms) conditions. Regarding the EEG observation, we observed N200-P300 wave activation in the middle occipital lobe and P300-N500 wave activation in the right frontal lobe and left motor cortex, which are associated with attention ERPs. Furthermore, delta (1–4 Hz) and theta (4–7 Hz) band powers in the right frontal lobe, as well as alpha (8–12 Hz) and beta (13–30 Hz) band powers in the left motor cortex were suppressed, whereas the theta (4–7 Hz) band powers in the middle occipital lobe were increased considerably in the attention task. Experimental results showed that the embodied body function influences human mental states and psychological performance under cognition attention tasks. These neural markers will be also feasible to implement in the real-time brain computer interface. Novel findings in this study can be helpful for humans to further understand the interaction between the brain and behavior in multiple target detection conditions in real life.

## 1. Introduction

Embodied cognition is a theoretical concept that assumes cognitive functions are closely related to the body and physical behavior [1,2]. In traditional cognitive model experiments, such as symbolic mental representations governed by logical and computational rules, the body only responds to information received by the senses. However, embodied cognition argues that the physicality of the body in action is not merely a vehicle for logical and computational process, but also the co-producer of the cognitive process [1]. In addition, the body reflects the state of the mind to some extent. For example, hand movements play a cognitive role in language development when speaking, while gestures and finger counting also help to express mathematical concepts. Embodied cognition is a highly flexible and complex mechanism, and it is difficult to explain this phenomenon with a single experiment, but it could be observed through experiments on cognition and physical behavior [1,3].

The theory of embodied cognition indicates that perception is based on the state and actions of the body, but research has also shown that there was no simple boundary between perception and action [4]. The cognitive state of the mind and the behavioral response of the body are the issues discussed in many studies [5]. Although psychology affects the body’s response, the degree of its influence is difficult to quantify through experiments. However, Gregg D’s [6] research showed that the change of mental state and the connection of the body and mind could be measured by the spectrum analysis of electroencephalographic (EEG) signal. For researchers studying situated cognition and sensorimotor function, embodied cognition is increasingly accepted as a viable theoretical choice [5,7,8].

Humans’ attention while focusing on a specific target would affect the neural activities of the human brain. Experiments designed with simple symbols, such as Go/No-Go tasks [9,10] and attention network tasks (ANT) [11,12,13] have been used in the study of mind–attention cognition tasks to investigate the neural activities of embodied mind cognition states. Although the neural activity of embodied mind cognition states can be understood through simple symbol experimental design, in the real world, focusing on a specific target is more complex than during an experiment in a laboratory. However, previous studies did not use a real-world environmental setting for the participants. Hence, the development of effective methods for measuring neural activities during mind–attention cognition tasks in real-world environments is critical. In the present study, we designed a novel embodied cognitive target detection experiment based on a real-world military scenario to explore the brain’s dynamic changes in the process of the mind and body’s response under a real-world setting target detection task.

This study is the first to adopt high-temporal-resolution wireless EEG recordings that are more suitable in the real word [14] for investigating the brain activities of individuals performing embodied mind cognition tasks in a military scenario by using independent component analysis (ICA) [15] and dipole source localization [16]. In addition, to further explore the interaction between the subject’s mental cognitive process and behavioral response under different commands and tasks, our experiment was designed for the participant to execute corresponding tasks after each command was issued, as compared with traditional ANT experiment settings with no-cue design [17,18]. The event-related potential (ERP) and event-related spectral perturbation (ERSP) techniques were used to investigate the neural activities of the brain when the participants were performing embodied mind cognition tasks under the experiment based on the military scenario. The ERSP reflects an event-related time/frequency state within the EEG signals and elucidates the extent to which underlying event-related synchronization or event-related desynchronization occurs [19]. Consequently, ICA and ERSP analysis provides a direct measure of the changes in the activity of the brain [15,19,20,21].

## 2. Materials and Methods

### 2.1. Participants

A total of 19 healthy participants (15 men and 4 women, aged 23 to 30 years) were recruited in this study [22,23]. All the participants were right-handed and had normal or corrected vision without a history of neurological or psychiatric diseases. This study was performed in accordance with the recommendations of the Institutional Review Board of National Chiao Tung University, Hsinchu, Taiwan, and was approved by the Research Ethics Committee of National Chiao Tung University, Hsinchu, Taiwan, under the protocol code NCTU-REC-108-085E. Informed consent included options to exclude these diseases and problems. Each participant had to sign the informed consent before the experiment.

### 2.2. Procedures

#### 2.2.1. Experimental Design

**Figure 1:**
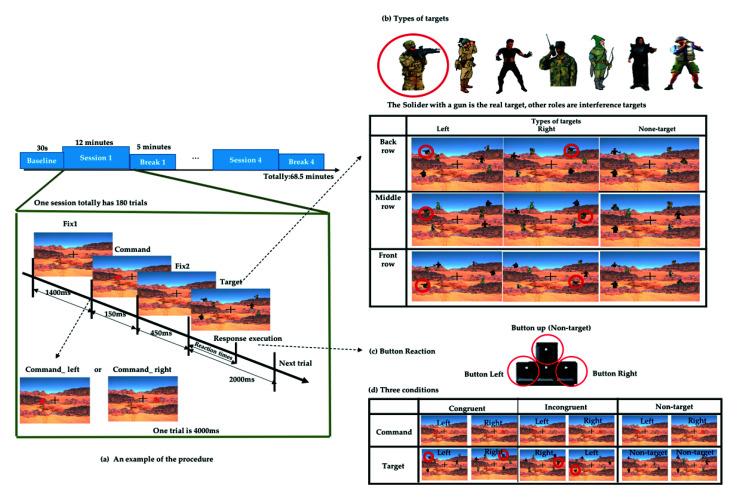
Experimental design of the simulated real-world target detection task based on a military scenario. (**a**) Schematic flow of the experiment. (**b**) Nine types of targets. (**c**) Response buttons on the keyboard. (**d**) Three different conditions.

The experiment was designed to simulate a real-world military shooting scenario using Unity (2018.3.6 version) software. Unity is a platform game engine developed by Unity Technologies, which creates three-dimensional (3D) and two-dimensional (2D) games, as well as interactive simulations and other experiences [24]. We used Unity to design the scenario and record the event mark and behavior data. The task scenario simulates the real environment for target search, and the real target was the image of a soldier wearing camouflage clothing with a black gun, and the interference object was the image of other characters wearing clothes of similar colors composed of gray, green, khaki, and black. As shown in Figure 1, the total duration of the experiment was 68.5 min, divided into 30 s of baseline (resting EEG). This was followed by 12 min sessions with a 5 min break between each session, and then followed by the next session with a total of four sessions and four breaks.

Each session included a total of 180 trials. In each trial, a fixation cross appeared in the center of the screen all the time. After a duration (1400 ms), a red asterisk command symbol appeared randomly on the left or right sides of the fixation cross in the center of the screen for 150 ms. After 450 ms, the target and interference object were presented, and the participant will search for the real target within 2 s and make an execution by pressing the corresponding arrow button after judging the previous command. We recorded the time from the appearance of the target until the subject executes the button as reaction times (RT), as shown in Figure 1a. When the real target appeared on the left side, the subject was supposed to press the “left” arrow button on the keyboard, whereas if the target appeared on the right side, the subject was supposed to press the “right” arrow button on the keyboard. When no specified target appeared, the subject was supposed to press the “up” arrow button, as shown in Figure 1c. In addition to the real target, there was other interfering targets. These targets with different characteristics formed a combination of 9 scenarios and appeared randomly in different trials, as shown in Figure 1b.

The command symbol appeared on the left or right side randomly, but the direction in which the target will did not necessarily correspond to it. According to the direction in which the command appears and the target appears, three different conditions were defined. When the command symbol and target appear on the same side, we define them as congruent conditions. Conversely, when the command symbol and target appear on different sides, we define them as incongruent conditions. If there are command symbols but no target appearing, we defined them as the nontarget conditions, as displayed in Figure 1d. These three conditions appeared in the same proportion in each session.

#### 2.2.2. EEG Acquisition and Preprocessing 

**Figure 2:**
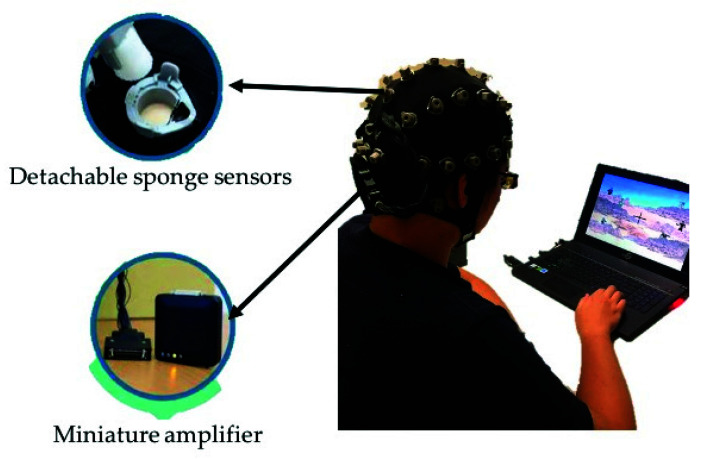
The wireless and wearable EEG system (St. EEGTM Vega) used in data acquisition.

EEG signals were collected from all healthy participants using a wireless EEG cap called St. EEG^TM^ Vega, as shown in Figure 2. St. EEG^TM^ Vega is manufactured by Artise Biomedical Co., Ltd., Taiwan. It features detachable water-based sponge sensors and a miniature amplifier, in which EEG signals were transmitted wirelessly through Bluetooth at a sampling rate of 500 Hz. St. EEG^TM^ Vega came with 35 sensors, including 32 recording electrodes, A1 and A2 reference electrodes, and FPz ground electrodes. All the 32 recording channels were located at the positions used in the International 10–20 systems.

**Figure 3:**
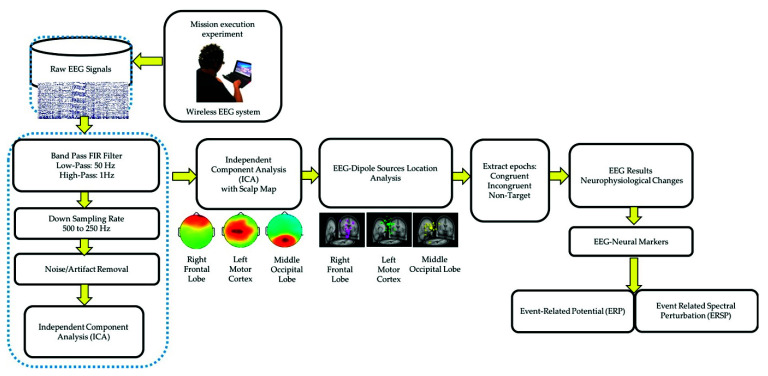
Flowchart of EEG signal analysis and preprocessing steps of acquired EEG signals.

All the EEG signals were evaluated using MATLAB R2012b (MathWorks Inc. Natick, MA, USA) and EEGLAB toolbox (10.2.2.4b version) [16,20]. The EEG signals were down sampled from 500 to 250 Hz. A 1–50 Hz finite impulse response bandpass filter was used to filter the EEG signals. ICA was performed to eliminate various artifacts, including muscle artifacts, eye movements, blinking artifacts, noise from indoor power lines, and environmental artifacts. ICA is an excellent computational method for the separation of blind sources in EEG signals [15,25]. An EEGLAB toolbox (10.2.2.4b version) and MATLAB R2012b (MathWorks Inc.) were used to perform ICA [16]. After performing ICA, EEG dipole source location analysis was conducted using DIPFIT2 routine functions of the EEGLAB toolbox (10.2.2.4b version) [15,16,25]. We then performed k-means clustering (K = 5) analysis using the aforementioned toolbox. Components with similar scalp maps, dipole locations, and power spectra were grouped in a cluster. We observed three consistent independent component scalp maps and dipole clusters, namely, right frontal lobe, left motor cortex, and middle occipital lobe, from the participants. We used these three clusters as the brain regions of interest to investigate the brain dynamics, in terms of ERP and ERSP, responsible for the interactions between the mind and body, while the participants were performing the embodied cognition experiment in the military scenario [16]. According to the event marker in EEG signals, each epoch was extracted from −500 to 2600 ms in the congruent, incongruent, and nontarget trials. In this study, each participant performed 720 trials under three conditions (congruent: 240 trials, incongruent: 240 trials, and nontarget: 240 trials). During the artifact removal procedure, poor-quality raw EEG signals were obtained in approximately 10% of the trials. Therefore, 10% of the EEG trials were removed to obtain clean EEG signals for ERP and ERSP analysis. Figure 3 displays the flowchart of the EEG signal analysis under the congruent, incongruent, and nontarget conditions.

### 2.3. Statistical Analyses

The behavioral data were collected using Unity software and were processed using MATLAB. For each participant, we categorized the response execution time corresponding to the three different trial conditions (as in Figure 1d) according to the event markers. Finally, we calculated the average and standard deviation of the 19 subjects under three conditions. We used one-way analysis of variance (ANOVA) and multiple comparison tests to determine whether the differences among the congruent, incongruent, and nontarget trials were significant (*p* < 0.05). 

In the EEG signal analysis, this study investigated the neural dynamics of the right frontal lobe, left motor cortex, and middle occipital lobe under congruent, incongruent, and nontarget conditions in the military-based embodied cognition scenario. ERP analysis was performed to determine the statistically (*p* < 0.01) significant differences among the congruent, incongruent, and nontarget conditions, based on the Wilcoxon signed-rank test. The yellow asterisks indicate pairwise significance between the congruent and incongruent conditions. Violet asterisks display pairwise significance between the incongruent and nontarget conditions. Green asterisks reveal pairwise significance between the congruent and nontarget. In the ERSP analysis, the statistically significant differences in the time and frequency domains were evaluated using the bootstrap method [16,26] at a significance threshold of *p* < 0.05 under congruent, incongruent, and nontarget trials over the right frontal lobe, left motor cortex, and middle occipital lobe. Furthermore, the average power spectral density (PSD) of EEG signals (1–50 Hz) was visualized under the congruent, incongruent, and nontarget conditions in the right frontal lobe, left motor cortex, and middle occipital lobe. Asterisks indicate a significant difference among the congruent, incongruent, and nontarget conditions in the Wilcoxon signed-rank test (* *p* < 0.05).

## 3. Results

### 3.1. Behavioral Results

**Figure 4:**
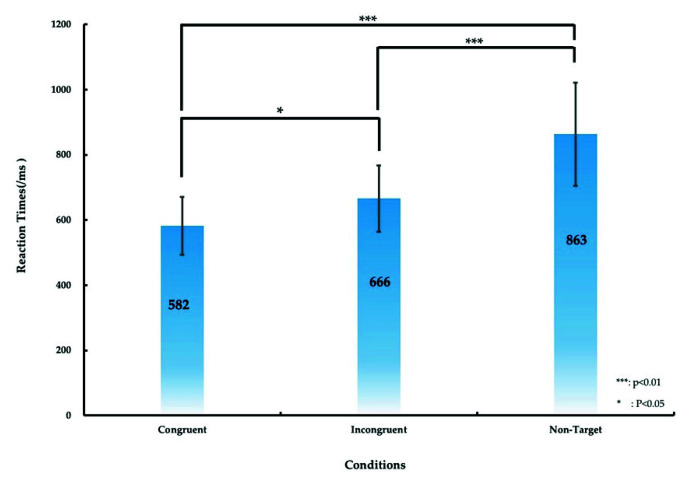
Differences in the RT under the congruent, incongruent, and nontarget conditions under the simulated real-world target detection task. The error bars denote the standard deviation. The asterisks denote the significance differences (* *p* < 0.05, *** *p* < 0.01) in the RT under the three conditions obtained using ANOVA1.

The significant difference in the RT between various conditions (the congruent, incongruent, and nontarget conditions) was calculated, as presented in Figure 4 and Table 1. Table 1 lists the RT of the participants under the aforementioned three conditions. The average RT was 582 ± 89, 666 ± 102, and 863 ± 158 ms under the congruent, incongruent, and nontarget conditions, respectively. We used ANOVA1 and post hoc comparisons to analyze the significant differences in RTs among the three conditions. ANOVA1 revealed significant differences in the RTs for the three conditions (F (2, 53) = 5.07, *p* < 0.01), and post hoc comparisons indicated that the RT under the congruent condition was significantly shorter than those under the incongruent and nontarget conditions (*p* < 0.05 and *p* < 0.01, respectively), as displayed in Figure 4. The RT under the incongruent condition was significantly shorter than that under the nontarget condition (*p* < 0.01), as illustrated in Figure 4.

### 3.2. EEG Results

#### 3.2.1. ICA Scalp Maps and Dipole Source Locations under Target detection

**Figure 5:**
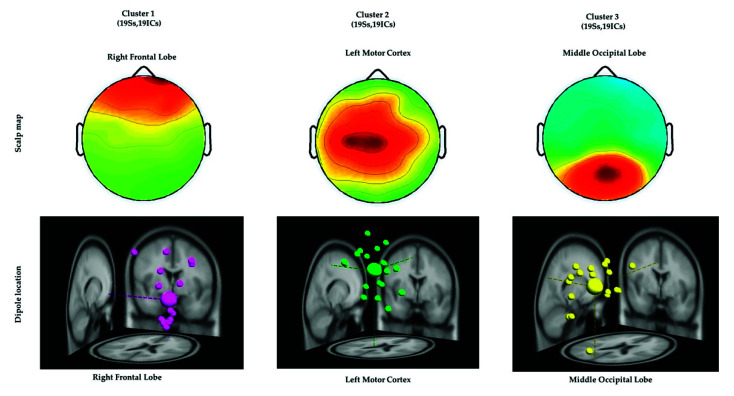
Three clusters of independent component scalp maps and dipole source locations at the right frontal lobe, left motor cortex, and middle occipital lobe of all the participants under the congruent, incongruent, and nontarget conditions. Each cluster reveals the average scalp maps and dipole locations for all the independent components within it. Cluster 1: right frontal lobe (N = 19). Cluster 2: left motor cortex (N = 19). Cluster 3: middle occipital lobe (N = 19), where N is the number of diploes projected in a cluster.

ICA was performed to isolate the EEG signals of independent brain regions from noise, such as eye activities and muscle activities that typically affect the analysis of EEG signals. After conducting ICA, we adopted the DIPFIT2 function to fit the dipoles in EEG signals by using the EEGLAB Toolbox and MATLAB [16,27,28]. The EEG dipoles were clustered using the k-means statistical analysis criteria (k = 3) [16,20]. The value of k was acquired by considering the potential number of dipoles that were related to the congruent, incongruent, and nontarget conditions. Furthermore, similar scalp maps and dipole locations were clustered into the same group for all the participants, as depicted in Figure 5. All three clusters and the Montreal Neurological Institute coordinates of their dipole source locations are presented in Table 2. These three brain regions directed the ERP and ERSP analysis of EEG signals associated with the embodied mind cognition state changes in the realistic military scenario, as described in the following sections.

#### 3.2.2. ERP Analysis 

##### ERP N500 and P300 Waves in the Right Frontal Lobe

**Figure 6:**
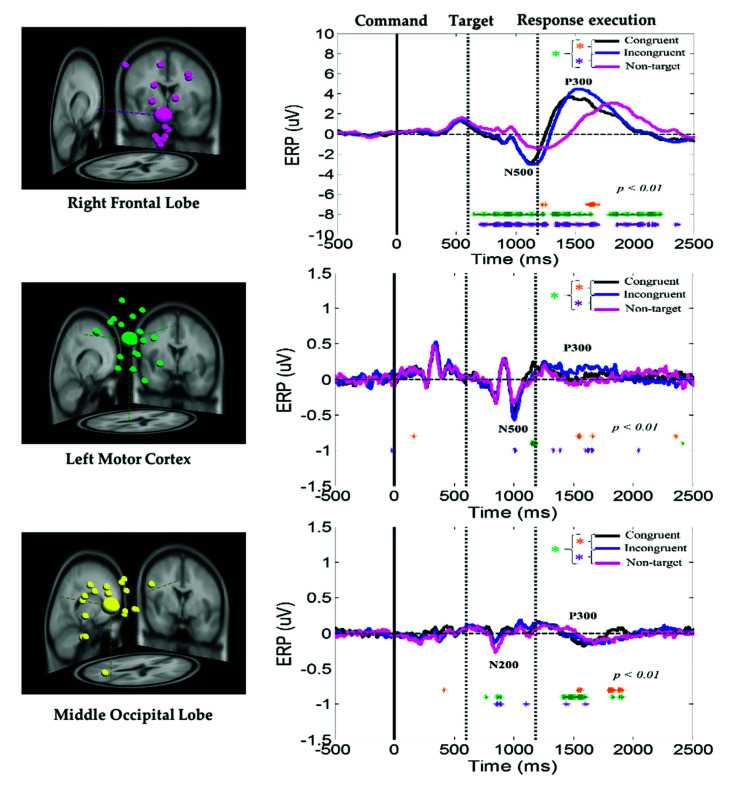
Average ERP under the congruent, incongruent, and nontarget conditions at the right frontal lobe, left motor cortex, and middle occipital lobe. Yellow asterisks indicate pairwise significance (* *p* < 0.01) between the congruent and incongruent conditions in the Wilcoxon signed-rank test. Violet asterisks denote pairwise significance (* *p* < 0.01) between the incongruent and nontarget conditions in the Wilcoxon signed-rank test. Green asterisks denote pairwise significance (* *p* < 0.01) between the congruent and nontarget conditions in the Wilcoxon signed-rank test.

The average ERP under the congruent, incongruent, and nontarget conditions at the right frontal lobe, left motor cortex, and middle occipital lobe of the brain are displayed in Figure 6. Peaks of the human visual-attention-related neural markers ERP N500 and P300 were observed in the right frontal lobe and left motor cortex under the congruent, incongruent, and nontarget conditions. In the right frontal lobe and left motor cortex of the brain, the amplitudes of the ERP N500 and P300 waves were considerably higher under the congruent and incongruent conditions than under the nontarget condition. In addition, in the right frontal lobe, the ERP N500 and P300 waves appear earlier under consistent and inconsistent conditions than under nontarget conditions. The aforementioned results revealed that, compared with nontarget conditions, participants detected the target earlier and caused stronger neural dynamics in the right frontal lobe in the congruent and incongruent conditions, while under nontarget conditions, the subject’s brain takes longer to detect the situation where no target image appears, and relatively showed weak nerve activity in the right frontal lobe. Furthermore, the ERP N200 and P300 waves were observed in the middle occipital lobe under the congruent, incongruent, and nontarget conditions. However, the amplitudes of these waves were higher in the right frontal lobe than in the left motor cortex and middle occipital lobe. The aforementioned ERP results indicate that the right frontal lobe is highly related to an embodied mind cognitive attention state. These ERP results show that body function influences human mental states.

#### 3.2.3. ERSP Analysis 

##### Delta and Theta Power Suppression in the Right Frontal Lobe

**Figure 7:**
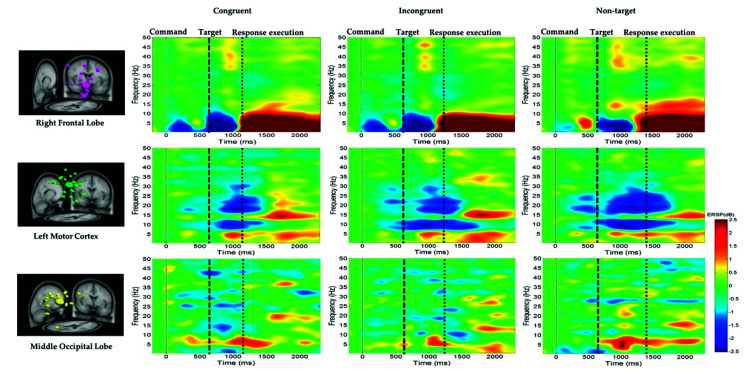
Average ERSP at the right frontal lobe, left motor cortex, and middle occipital lobe under the congruent, incongruent, and nontarget conditions. The first magenta line denotes the command onset. The second black dashed line reveals the target. The third black dashed line represents the response execution after target detection. The ERSP difference is significant at *p* < 0.05. The colored bars indicate the scale of ERSP.

The average ERSP under the congruent, incongruent, and nontarget conditions in the right frontal lobe, left motor cortex, and middle occipital lobe of the participants are shown in Figure 7. For the right frontal lobe, the embodied cognition attention-related EEG activity of delta (1–4 Hz), theta (4–7 Hz), and alpha (8–12 Hz) powers from 600 to 1200 ms were lower under the congruent and incongruent conditions than under the nontarget conditions. However, after hand response, in the right frontal lobe, the movement of the hand-related EEG activity in the delta (1–4 Hz), theta (4–7 Hz), and alpha (8–12 Hz) band powers from 1200 to 2600 ms was higher in the nontarget conditions than in the congruent and incongruent conditions.

##### Mu Rhythm Suppression in the Left Motor Cortex

Figure 7 also indicates that the alpha (8–12 Hz) and beta (13–30 Hz) band powers or mu rhythms at the left motor cortex were suppressed to a greater extent in the nontarget condition than in the congruent and incongruent conditions after target onset. This expected alpha and beta power suppression is associated with embodied cognition attention state and hand movement. Figure 7 also indicates that the theta (4–7 Hz) band power at the middle occipital lobe was higher in the nontarget condition than in the congruent and incongruent conditions after target onset. The beta power (13–30 Hz) at the occipital lobe was lower in the congruent condition than in the other conditions. The EEG power activity changes were related to an embodied cognition attention state.

#### 3.2.4. EEG PSD Analysis

**Figure 8:**
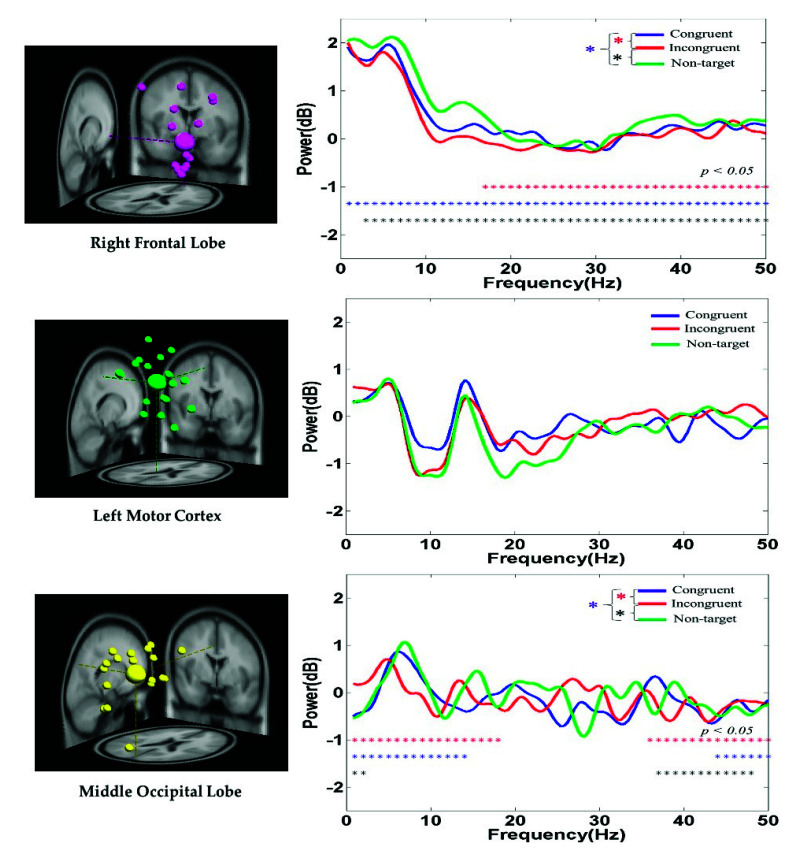
Average PSD of EEG signals at the right frontal lobe, left motor cortex, and middle occipital lobe under the congruent, incongruent, and nontarget conditions. Asterisks denote the significant differences among the congruent, incongruent, and nontarget conditions in the Wilcoxon signed-rank test (*** *p* < 0.05).

The average PSD of the EEG signals in the right frontal lobe, left motor cortex, and middle occipital lobe of the participants under the congruent, incongruent, and nontarget conditions are displayed in Figure 8. At the right frontal lobe, the hand-related EEG PSD activity in the delta (1–4 Hz), theta (4–7 Hz), and alpha (8–12 Hz) band powers was higher in the nontarget condition than in the congruent and incongruent conditions after hand response. Figure 8 indicates that the alpha (8–12 Hz) and beta (13–30 Hz) band powers at the left motor cortex were suppressed to a greater extent in the nontarget condition than in the congruent and incongruent conditions. The aforementioned findings are related to the movement of the right hand. These PSD results show that embodied body function influences human mental states. Figure 8 also indicates that the theta (4–7 Hz) band power at the middle occipital lobe was higher in the nontarget condition than in the congruent and incongruent conditions. This result is attributed to the cognition visual stimulation in the occipital lobe.

## 4. Discussion

In the present study, we used a wireless EEG system to investigate neural activities when performing embodied mind cognition tasks in a military scenario through ICA and dipole source location analysis. High-temporal-resolution EEG signals were used to observe the neural markers of embodied cognition in the right frontal lobe, left motor cortex, and middle occipital lobe under congruent, incongruent, and nontarget conditions in a military setting. By combining behavioral methods and ICA, this study identified the neural EEG markers of embodied cognitive attention states in the right frontal lobe, left motor cortex, and middle occipital lobe.

### 4.1. Behavior Outcomes When Performing Embodied Cognition Tasks

The RT in the congruent condition was shorter than those in the incongruent and nontarget conditions (Figure 4). This result reveals that all the participants paid more focus to searching for a real target under the congruent condition than under other conditions. We compared the results obtained under the congruent and incongruent conditions with those of a previous study on cognition attention tasks [29]. The aforementioned behavioral findings are consistent with those of a previous study on cognition attention tasks [29]. In this study, in the nontarget condition, all the participants exhibited a long RT because they exerted some effort in searching for the target (soldier with a gun). This finding revealed that human mental state influenced body function and performance in cognition attention-related tasks.

### 4.2. EEG–ERP N500 and P300 Waves in the Right Frontal Lobe 

In this study, peaks of the embodied cognitive attention-related EEG neural markers ERP N200, N500, and P300 were detected in the right frontal lobe and left motor cortex under the congruent, incongruent, and nontarget conditions (Figure 6). These ERP waves had a higher amplitude in the congruent and incongruent conditions than in the nontarget condition. The ERP N500 and P300 waves had a higher amplitude in the right frontal lobe than in the left motor cortex and middle occipital lobe. These ERP neural markers revealed that the right frontal lobe was related to embodied cognition attention. Most previous cognition attention studies have reported N200 and P300 waves in the frontal lobe [11,30,31,32,33]. Furthermore, ERP-N200 and P300 waves have been proven to be related to cognition attention in the frontal lobe of the brain. Furthermore, ERP-N200 and P300 waves have been examined under human inhibitory control [30,34]. N500 was previously found to be related to the semantic incongruities in audio perception [35]. In this study, for the first time, we investigated the ERP neural markers of the frontal lobe (N500), left motor cortex (N500 and P300), and middle occipital lobe (N200 and P300) of the brain. The ERP neural markers and short behavioral RTs under the congruent condition revealed that all the participants were more likely to pay attention to the target appearing in the congruent condition of a military scenario. Additionally, the subjects need to spend more time and thinking when the command and the execution of the task are not in conformity. These ERP findings show that embodied body function influences mental states under cognition attention-related tasks.

### 4.3. Delta, Theta, and Alpha Power Suppression in the Right Frontal Lobe

The average ERSP results indicated that delta, theta, and alpha power suppression occurred in the right frontal lobe, as displayed in Figure 7. The cognition attention-related EEG neural activity of the delta, theta, and alpha band powers were lower in the congruent condition than in the incongruent and nontarget conditions. Cognition attention-related studies have revealed that theta and alpha band power suppression is related to human cognition attention in the frontal lobe of the brain [36,37,38,39]. Furthermore, in the right frontal lobe of the brain, the movement of the hand-related EEG powers of the delta, theta, and alpha bands from 1200 to 2600 ms were higher in the nontarget condition than in the congruent and incongruent conditions. The aforementioned ERSP findings reveal that the frontal lobe is related to the hand response and cognition attention.

### 4.4. Alpha and Beta Power Suppression in the Left Motor Cortex

We measured the spectral changes in the EEG power, such as the suppression of alpha and beta powers, at the left motor cortex (Figure 8). Alpha power suppression was considerably higher in the nontarget condition than in the congruent and incongruent conditions. This result reveals that the left motor cortex is related to cognition attention and hand movement. Moreover, the alpha and beta band power suppression in the motor cortex is consistent with the results of previous studies conducted on healthy individuals under walking conditions [40,41,42,43]. The theta band power was considerably higher in the nontarget condition than in the congruent and incongruent conditions in the middle occipital lobe, and the beta power in the middle occipital lobe was lower than that in the other examined brain regions (Figure 8). A classroom attention study reported an increase in the delta and theta EEG powers in the occipital lobe and a decrease in the beta power in the occipital lobe [44]. These results reveal that the occipital lobe is related to an embodied cognition attention task and visual stimulation under a military scenario.

### 4.5. The Limitations of This Approach

Some of the previous studies explored how aging affects the influence of embodiment on mental representations. They examined age-related differences in mental imagery, motor imagery, and action observation [45,46]. The limitation of the present study is that normal university students have participated in this experiment. In future work, we will invite children and older adults to extend our research. Secondly, the experimental scene in the current study was the simulated real-world target detection task based on a military scenario, which uses only two-dimensional figures and may not be the most realistic setting for our subjects. Future work may construct an even more realistic environment in virtual reality (VR) to increase the impact of target searching. Designing a new scenario for imaging the human brain during target searching, such as in a virtual reality environment, will provide further insight regarding embodied cognition.

## 5. Conclusions

This study is the first to use a wireless EEG system to investigate the brain dynamics of human attention in an embodied military scenario by using ICA and dipole source location analysis. In this study, considerable brain activity features stemming from cognition and attention had been obtained from a target detection task in a novel military scenario. The behavioral results of this study revealed that the RT in the congruent condition was considerably shorter than those in the incongruent and nontarget conditions. Furthermore, the EEG results revealed the existence of three human attention-related ERP markers, namely, ERP-N200, N500, and P300, in the right frontal lobe, left motor cortex, and middle occipital lobe of the brain. We examined the embodied attention-related power spectral change, including the delta and theta power suppression in the right frontal lobe, as well as the alpha and beta power suppression in the left motor cortex in an attention task. The theta power was higher in the middle occipital lobe than in the other brain regions. Our findings demonstrate the feasibility of studying human target detection brain activity in a simulated real-world condition. In addition, we detected the presence of the N500 marker in a visual-based cognitive study. The obtained EEG results can be useful for researchers to understand the interaction between the brain and human behavior in multiple target detection tasks.

## Figures and Tables

**Table 1:** RT of the participants, including the average and standard deviation of the RT under the congruent, incongruent, and nontarget conditions.

Subjects	Congruent RT (ms)	Incongruent RT (ms)	Nontarget RT (ms)
1	635	725	887
2	696	753	1030
3	454	665	830
4	492	535	723
5	551	697	991
6	665	704	902
7	535	631	716
8	600	799	983
9	456	648	766
10	580	600	664
11	486	537	754
12	645	630	843
13	622	693	842
14	501	614	813
15	759	851	1199
16	584	637	950
17	547	584	816
18	655	757	944
19	598	592	744
Avg ± SD	582 ± 89	666 ± 102	863 ± 158

**Table 2:** Three independent component clusters in the brain and the Montreal Neurological Institute coordinates of their dipole source locations under the congruent, incongruent, and nontarget conditions.

Component Clusters	Side	Brain Regions	MNI Coordinates (mm)			Cluster Size (Voxels)
			X	Y	Z	
1	Right	Frontal Lobe	7	57	−13	6
2	Left	Motor Cortex	−9	−26	54	9
3	Middle	Occipital Lobe	−2	−80	39	12

MNI: Montreal Neurological Institute.

## Data Availability

The EEG datasets generated for this study are available on request to the corresponding author.
